# Association Between Euthyroidism and Muscular Parameters in Adults with an Excess of Fat Mass: A Preliminary Study

**DOI:** 10.3390/healthcare13030241

**Published:** 2025-01-24

**Authors:** Francesca Greco, Luciana Sicilia, Giuseppe Seminara, Stefano Iuliano, Vera Tocci, Antonio Brunetti, Antonio Aversa, Luigi Di Luigi, Paolo Sgrò

**Affiliations:** 1 Department of Movement, Human and Health Sciences, University “Foro Italico” of Rome, 00135 Rome, Italy; 2 Department of Clinical and Experimental Medicine, Magna Græcia University of Catanzaro, 88100 Catanzaro, Italygiuseppe.seminara@studenti.unicz.it (G.S.);; 3 Department of Health Sciences, Magna Græcia University of Catanzaro, 88100 Catanzaro, Italytocci.vera@gmail.com (V.T.);

**Keywords:** thyroid hormone, muscle mass, fat mass, muscle strength, muscle performance

## Abstract

Background/Objectives: Thyroid hormones (THs) are correlated with body composition regardless of the presence of thyroid dysfunctions, whereas little is known regarding THs’ influence on muscular fitness components. This cross-sectional study investigated whether THs may affect muscular parameters in adults who are overweight and affected obesity. Methods: One hundred and five volunteers (65 females and 40 males, BMI: 33.5 (8.0) kg/m^2^) in an euthyroid state were enrolled. Body composition was evaluated using bioelectrical impedance analysis. Muscular parameters of interest were grip strength—evaluated using the handgrip test (HG) and muscular performance –evaluated using the 10-repetition chair-stand test (CST). Free-triiodothyronine (FT3), free-thyroxine (FT4) and their ratio (FT3/FT4) were evaluated to assess thyroid function. Results: In the female group, the Pearson linear regression analysis revealed a positive correlation between FT3 and HG (R = 0.261, *p* = 0.036) and a negative correlation between FT3/FT4 and CST (R= −0.266, *p*= 0.032). In the male group, a positive correlation between FT3 and skeletal muscle mass was found (R = 0.354, *p* = 0.025). No correlations were found between THs of interest and adiposity indexes (BMI, fat mass) in either group (*p* > 0.05). Conclusion: FT3 and FT3/FT4 ratio levels in euthyroidism may influence muscular parameters differently in females and males affected by an excess of fat mass. More research is needed to understand the mechanisms behind this correlation and whether THs could be a potential biomarker for muscle-related parameters.

## 1. Introduction

Thyroid hormones are involved in several metabolic processes (e.g., thermogenesis, lipid and glucose metabolism) [1,2] representing an important part of the endocrine system. An excess of thyroid functioning (i.e., hyperthyroidism) promotes a hypermetabolic state, whereas reduced thyroid hormone levels (i.e., hypothyroidism) are associated with hypometabolism [1,2]. Abnormalities in the hypothalamic–pituitary axis may occur as a cause or consequence of obesity although this bidirectional link is complex and not yet fully understood [3].

Obesity is responsible for increased serum concentrations of thyrotropin (TSH) and slightly higher than normal free plasma levels of free triiodothyronine (FT3) [4]. The slight increase in FT3 levels may represent a compensatory mechanism as an adaptive thermogenic phenomenon [5]. Moreover, thyroid hormones are closely correlated with changes in body mass index and body composition regardless of thyroid dysfunctions [3,6]. Among several complications (e.g., cardiovascular diseases, and metabolic problems), obesity also seems to impair skeletal muscle function [7,8] which is known to represent an important health-related component [9]. Therefore, obesity-related changes in muscular quantity and quality may be clinically important. Indeed, the coexistence of reduced muscular function together with an excess of fat mass may occur in obesity [10].

Thyroid hormones regulate skeletal muscle development, as well as its contractile activity, metabolism, and regeneration processes [11,12], thus, making skeletal muscle a major site of thyroid hormone signaling [11]. Based on this, as reported by a recent systematic review, the relationship between thyroid hormones and muscular parameters in different euthyroid populations was investigated [13]. Specifically, it has been reported that a preserved skeletal muscle mass within a framework of excess body mass directly influences thyroid function [14]. Moreover, a significant association between thyroid hormones and muscle strength in overweight and obese individuals has been found [15]. Since the skeletal muscle is an important site for several metabolic processes (e.g., glucose and lipid metabolism) an interaction between thyroid functioning and muscular components is probable. It should be noted that the conversion of thyroxine (T4) to T3 occurs in the skeletal muscle due to the role of the type II iodothyronine deiodinase activity [12]. Therefore, it may be that a good muscular fitness status could relate to thyroid hormone levels in individuals with excess fat mass. The study aimed to examine the association between muscular parameters and thyroid hormone levels among individuals with obesity and who are overweight.

## 2. Material and Methods

### 2.1. Study Design

This study adopted a cross-sectional study design. Participants were enrolled at the Endocrinological Unit of the “Renato Dulbecco” University Hospital, Catanzaro, Italy. All data collection was done at the baseline examination which consisted of general information, medical history, assessing body composition, muscular fitness components, physical activity levels and thyroid hormones. All participants gave written informed consent before enrolment and experimental procedures complied with the Declaration of Helsinki.

### 2.2. Participants

One hundred and fifteen participants were recruited. Of these, one hundred and five (40 M, 65 F) accomplished all the experimental procedures. The characteristics of participants who completed the intervention protocol are reported in Table 1.

Inclusion criteria were males and females aged between 18 and 64 years old, with a BMI ≥ 25 kg/m^2^.

Exclusion criteria were the use of levothyroxine, antithyroid drugs, a history of thyroid surgery, thyroid radiation, pregnancy, any history of endocrinological diseases (e.g., hypo or hyperthyroidism), renal dysfunctions and chronic inflammatory diseases.

### 2.3. Anthropometric and Body Composition Evaluations

- Height and body mass were measured using a stadiometer with a weighting station to the nearest 0.1 cm and 0.1 kg, respectively (SECA, Intermed S.r.l., Milano, Italy). Body mass index (BMI) was calculated by dividing body mass (kg) by the square of height (m^2^).
- Body composition was measured using a bioelectrical impedance method (BIA 101 BIVA^®^ PRO AKERN s.r.l., Pisa, Italy) in the morning. Whole-body impedance resistance and reactance were obtained and recorded when stable. Variables of interest were percentage of fat mass (pFM), fat-free mass (FFM) and skeletal muscle mass (SMM).

### 2.4. Muscular Fitness Assessments

A handgrip strength test (HG) was performed to evaluate the maximum isometric strength of the hand and forearm muscles using a Jamar hydraulic hand dynamometer [16]. Participants were instructed to contract as hard as possible for three seconds maintaining the elbow at right angles. Three trials were performed on each hand and the mean of right and left hands was used for the analysis.

The chair stand test (CST) evaluated lower limb muscular performance. Participants were asked to stand up and sit down ten consecutive times on a standard chair without armrests in the shortest possible time. The time (s) to execute the task was recorded with a stopwatch and used for the analysis [8].

### 2.5. Physical Activity Levels

The Global Physical Activity Questionnaire (G-PAQ) was administered to obtain information on physical activity participation in three domains (activity at work, travel to and from places, and recreational activities) [17]. The total amount of physical activity in all domains across a typical week (METs/week) was used for the analysis.

### 2.6. Thyroid Hormone Assays

Blood samples were collected in the morning and analyzed at the “Renato Dulbecco” University Hospital, Catanzaro, Italy. A chemiluminescence immune analyzer method determined TSH, FT3 and FT4 (ADVIA Centaur XP Immunoassay System, Siemens, Malvern, PA, USA). As determined by internal validation, normal values were as follows: TSH: 0.55–4.78 ulU/mL; FT3: 2.3–4.2 pg/mL; FT4: 0.7–1.76 ng/dL. The FT3 to FT4 ratio was calculated to assess peripheral sensitivity (peripheral thyroxin deiodination) [18].

### 2.7. Statistical Analysis

All data are presented as mean values ± standard deviation (SD) and median (interquartile range, IQR). The normal distribution of the dependent variables was tested using the Shapiro–Wilk test. Differences at baseline between males and females were evaluated with an unpaired *t*-test when variables were normally distributed. The corresponding non-parametric test was performed when variables were not normally distributed. Correlation between thyroid hormones as dependent variables (FT3, FT4, FT3/FT4) and muscular components (SMM, HG and CST) as independent 1 was conducted with Pearson linear regression analysis. The level of significance was set at *p* < 0.05. Statistical analysis was run with IBM^®^ SPSS statistics software version 23.0 (SPSS Inc., Chicago, IL, USA).

## 3. Results

There was a greater proportion of females in the whole sample (61.9%). The median age for both sexes was 44.0 (28.5) years and the median BMI of 33.5 (8.0) kg/m^2^. A total of 33.3% of the participants were in the range of 25 ≤ BMI ≥ 30 kg/m^2^ and 66.7% with BMI > 30 kg/m^2^. Physical activity levels assessed with G-PAQ were statistically significantly higher in the male group than the female one (540.0 (2218) vs. 360.0 (1300) METs/week, *p* = 0.031).

Table 1 summarizes the general, anthropometric, hormonal and muscular parameters of the enrolled participants expressed as mean ± SD and median (IQR). A correlation matrix for all comparisons is reported in Table 2 for males and Table 3 for females.

**Figure 1:**
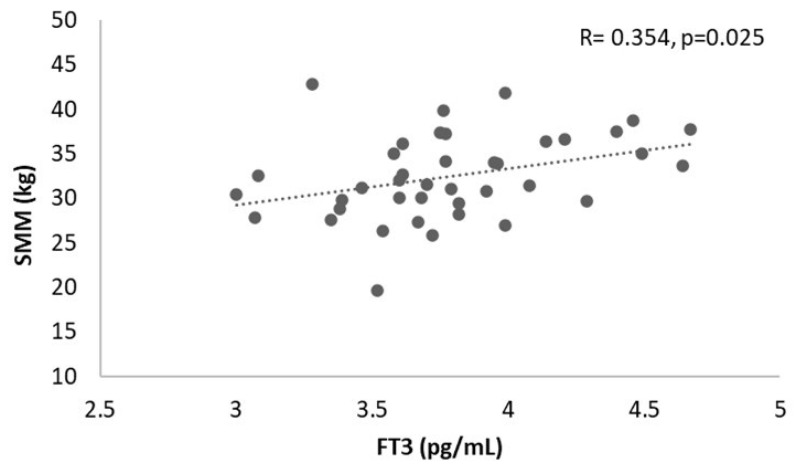
Correlation analysis between SMM and FT3 in the male sample. SMM, skeletal muscle mass; FT3, free triiodothyronine.

In the male group, a positive correlation between FT3 and SMM was found (R = 0.354, *p* = 0.025) (Figure 1). No correlations for the other variables of interest were found (*p* > 0.05).

**Figure 2:**
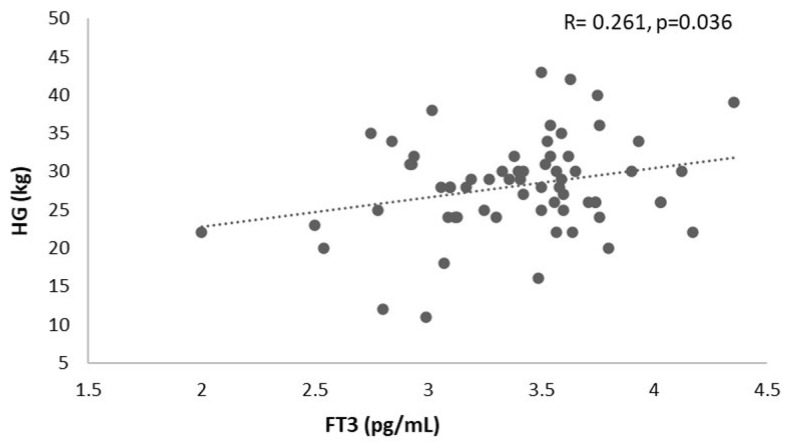
Correlation analysis between HG and FT3 in the female sample. HG, handgrip strength; FT3, free triiodothyronine.

**Figure 3:**
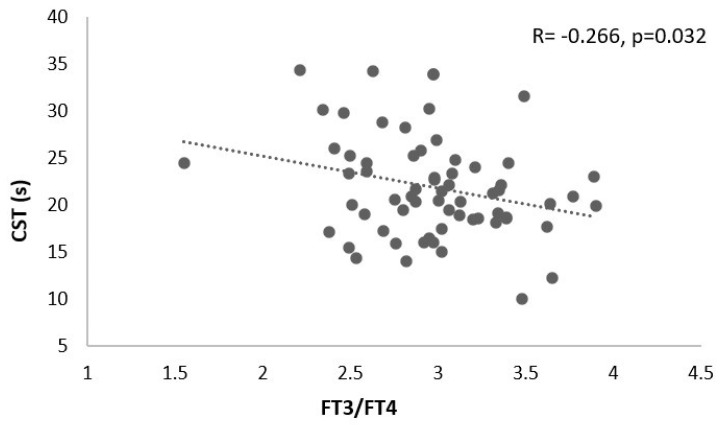
Correlation analysis between CST and FT3/FT4 in the female sample. CST, chair stand test; s, seconds.

In the female group, a positive correlation between FT3 and HG (R = 0.261, *p* = 0.036) (Figure 2) and a negative correlation between FT3/FT4 and CST (R = −0.266, *p* = 0.032) was found (Figure 3). No correlations for the other variables of interest were found (*p* > 0.05).

## 4. Discussion

This study investigated whether thyroid hormones could be considered biomarkers associated with muscular components in individuals affected by obesity. Thyroid hormones play a crucial role in skeletal muscle development, suggesting that thyroid function may influence muscle mass and strength, representing significant health-related indicators [9,11,12]. Our results revealed that FT3 and FT3/FT4, but not FT4, may affect muscular components. Specifically, depending on the type of muscular index analyzed (quantitative or functional), the association differed between males and females. As type II iodothyronine deiodinase (DIO2) activity is expressed in skeletal muscle cells [12,19], the role of skeletal muscle in local and systemic T3 production could be expected. Indeed, in line with this assumption, we found correlations only with FT3 and FT3/FT4 and not with FT4 levels. When analyzing muscle quantity, our results revealed a positive correlation only in the male sample, with FT3 levels positively correlating with SMM. The significant difference in FT3 between men and women could be referred to the different amounts of muscular tissue and, thus, in terms of a higher FT4 to FT3 conversion. This result aligns with a previous study conducted on overweight and obese individuals revealing an association with muscular parameters derived from body composition analysis only in males [14]. This result may depend on the difference in body composition between sexes as males exhibit a higher amount of skeletal muscle than females [20]. However, our results did not find a correlation between FT3/FT4 and SMM as revealed by previous investigations reported in a recent systematic review conducted on euthyroid populations [13]. De Pergola et al. [5] suggested an increased conversion from FT4 to FT3 in euthyroidism which could be related to increased deiodinase activity in obesity. The FT3/FT4 ratio may be considered a marker of dynamic DIO2 activity, reflecting several factors, including TSH influence and metabolic changes (e.g., insulin resistance, metabolic syndrome) in obesity [5]. Therefore, it may be that the peripheral sensitivity could differ between non-obese and euthyroid-obese individuals. Further investigations are needed to clarify the possible mechanism behind the association between thyroid hormones and muscle mass. Moreover, when referring to muscular function parameters (i.e., handgrip strength, HG and lower limb performance, CST) no correlations with thyroid hormones were found in the male sample. Instead, a positive correlation between FT3 levels and HG and a negative correlation between FT3/FT4 and CST in the female sample was observed. It has been previously reported that low levels of FT3 were associated with reduced physical performance in older adults [21,22]. Moreover, a longitudinal population-based cohort study of high-normal serum FT3 levels and higher FT3/FT4 ratios significantly predicted the change in HG among middle-aged and older euthyroid individuals [23]. Therefore, this confirms that FT3 and FT3/FT4 may represent an important indicator related to muscular function. It is worth mentioning that the main active form of thyroid hormones is FT3, which is essential for skeletal muscle development and regeneration. Local conversion of T4 to active T3 in skeletal muscle is predominantly mediated by DIO2 [12]. As deiodinase activity reflects intracellular T3 concentration [11], a reduction in muscular function is related to higher FT4 levels. Consequently, it can be hypothesized that a lower FT3/FT4 ratio may be a sign of decreased physical function and reduced systemic deiodinase activity. According to our results, it may be that thyroid hormones act differently depending on which aspect of the muscle is considered. Specifically, ‘quantitative’ data (i.e., SMM) seem to influence men, whereas functional data (i.e., HG and CST) seem to affect women. This may also depend on the different physical fitness status and physical activity levels with males reporting higher values than females. A graphical conceptual summary is reported in Figure S1. To the best of our knowledge, this is the only study that, at the same time, analyzed quantitative and qualitative indexes of muscular function in a population who are overweight and affected by obesity. It is worth noting that the muscular parameters analyzed in the present study are of clinical relevance as referred to sarcopenia and muscle quality assessments in the obese population [8,10].

## 5. Limitations of the Study

The authors are aware of some study limitations. Due to the cross-sectional design of the study, a causal explanation cannot be made. Moreover, as different “obesities” exist, using BMI as classification without distinguishing between different subcategories may not be enough. Despite the euthyroid condition of our participants, the lack of thyroid antibody analysis may have masked the presence of potential occult thyroidal conditions. The small sample size (the majority of which were males) considered, as well as the lack of a normal-weight control group and more comprehensive clinical information including metabolic (e.g., serum lipids) parameters, dietary information and muscular enzymes (e.g., creatine kinase, lactose dehydrogenase and aldolase), does not allow us to clarify the potential mechanisms behind this association, which merits further attention.

## 6. Conclusions

Levels of FT3 and the FT3/FT4 ratio in the euthyroidism state may influence muscular parameters in females and males affected by an excess of fat mass. Specifically, skeletal muscle mass values correlated only in the male group, whereas handgrip and chair stand results correlated only in the female group. More research is needed to understand the mechanisms behind this correlation. Future studies are needed to clarify whether thyroid hormones in a euthyroid state could be considered as a biomarker associated with muscular fitness in individuals who are overweight and affected by obesity.

## Figures and Tables

**Table 1:** Characteristics of the sample (n = 105) reported by sex.

Variable	N = 40 M	N = 65 F	M vs. F *p*-Value *
Age (years)	44.5 (27.8)	41.0 (29.5)	0.550
Body mass (kg)	95.3 (24.3)	84.0 (16.2)	<0.01
Body Mass Index (kg/m^2^)	33.0 (10.6)	34.0 (7.5)	0.611
Fat Free Mass (kg)	65.4 ± 9.2	48.8 ± 5.4	<0.01
Skeletal Muscle Mass (kg)	32.5 ± 4.8	24.0 ± 3.3	<0.01
Fat Mass (%)	34.2 ± 8.4	43.6 ± 6.4	<0.01
TSH (ulU/mL)	1.82 (1.69)	2.19 (1.68)	0.145
FT3 (pg/mL)	3.78 ± 0.41	3.40 ± 0.43	<0.01
FT4 (ng/dL)	1.19 ± 0.14	1.16 ± 0.17	0.235
FT3/FT4	3.20 ± 0.44	2.97 ± 0.43	<0.01
Handgrip strength (kg)	44.0 ± 11.5	28.1 ± 6.2	<0.01
Chair Stand Test (seconds)	18.7 ± 3.5	21.9 ± 5.4	<0.01

* Unpaired *t*-test or the corresponding non-parametric test between M and F. Data are reported as mean ± standard deviation and median (interquartile range). N, sample; M, males; F, females; TSH, thyroid-stimulating hormone; FT3, free triiodothyronine; FT4, free thyroxine.

**Table 2:** Correlation matrix for all comparisons in males (n = 40).

Variable	FT3 (pg/mL)		FT4 (ng/dL)		FT3/FT4	
	R	*p*-Value	R	*p*-Value	R	*p*-Value
Body mass (kg)	0.265	0.099	0.287	0.073	−0.040	0.805
Body Mass Index (kg/m^2^)	0.216	0.181	0.235	0.144	−0.033	0.842
Fat Free Mass(kg)	0.245	0.128	0.180	0.267	0.022	0.893
Skeletal Muscle Mass (kg)	0.354	0.025 *	0.154	0.343	0.108	0.509
Fat Mass (%)	0.185	0.253	0.271	0.091	−0.077	0.637
TSH (ulU/mL)	−0.013	0.935	0.020	0.904	−0.039	0.810
Handgrip strength (kg)	0.005	0.973	0.178	0.273	−0.160	0.325
Chair Stand Test (s)	−0.073	0.656	−0.172	0.289	0.085	0.601

* Denotes statistical significance; TSH, thyroid-stimulating hormone; FT3, free triiodothyronine; FT4, free thyroxine.

**Table 3:** Correlation matrix for all comparisons in females (n = 65).

Variable	FT3 (pg/mL)		FT4 (ng/dL)		FT3/FT4	
	R	*p*-Value	R	*p*-Value	R	*p*-Value
Body mass (kg)	−0.042	0.738	−0.028	0.824	−0.013	0.915
Body Mass Index (kg/m^2^)	−0.119	0.346	0.014	0.912	−0.126	0.318
Fat Free Mass(kg)	0.030	0.815	−0.008	0.949	0.038	0.763
Skeletal Muscle Mass (kg)	0.018	0.889	−0.002	0.990	−0.010	0.938
Fat Mass (%)	−0.006	0.959	−0.027	0.830	0.027	0.831
TSH (ulU/mL)	0.049	0.700	−0.198	0.115	0.238	0.056
Handgrip strength (kg)	0.261	0.036 *	0.195	0.120	0.026	0.838
Chair Stand Test (s)	−0.047	0.709	0.243	0.051	−0.266	0.032 *

* Denotes statistical significance; TSH, thyroid-stimulating hormone; FT3, free triiodothyronine; FT4, free thyroxine.

## Data Availability

Dataset will made available upon request.

## Supplementary Materials

The following supporting information can be downloaded at: https://www.mdpi.com/article/10.3390/healthcare13030241/s1, Figure S1: Graphical Conceptual Summary.
