# Supplementary figures and images for: Association Between Euthyroidism and Muscular Parameters in Adults with an Excess of Fat Mass: A Preliminary Study
Source: Healthcare (Basel). 2025 Jan 24;13(3):241. doi: 10.3390/healthcare13030241 (PMC11817674; doi:10.3390/healthcare13030241)

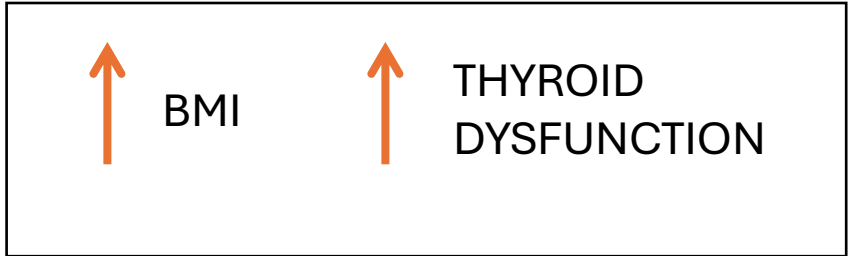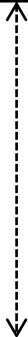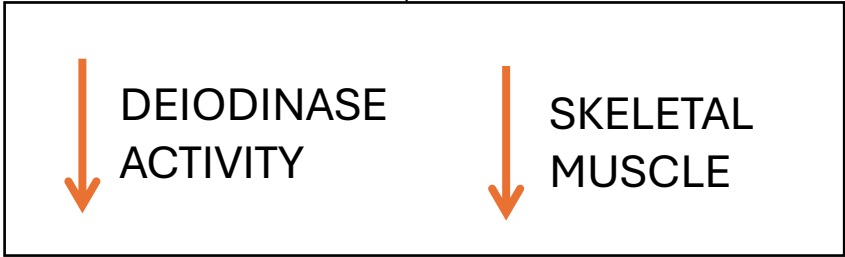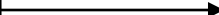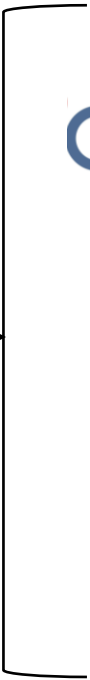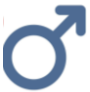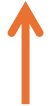

MUSCLE MASS

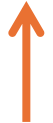

FT3

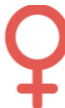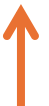

FUNCTION

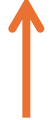

FT3

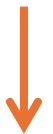

FT3/FT4

Supplement: Supplementary file 1 [file healthcare-13-00241-s001.zip › Figure S1 Graphical Conceptual Summary.pdf]
